# Satellite RNAs: emerging players in subnuclear architecture and gene regulation

**DOI:** 10.15252/embj.2023114331

**Published:** 2023-08-01

**Authors:** Kensuke Ninomiya, Tomohiro Yamazaki, Tetsuro Hirose

**Affiliations:** ^1^ Graduate School of Frontier Biosciences Osaka University Suita Japan; ^2^ Institute for Open and Transdisciplinary Research Initiatives (OTRI) Osaka University Suita Japan

**Keywords:** membraneless organelle, noncoding RNA, satellite DNA, stress response, T2T genome, Chromatin, Transcription & Genomics, RNA Biology

## Abstract

Satellite DNA is characterized by long, tandemly repeated sequences mainly found in centromeres and pericentromeric chromosomal regions. The recent advent of telomere‐to‐telomere sequencing data revealed the complete sequences of satellite regions, including centromeric α‐satellites and pericentromeric HSat1–3, which together comprise ~ 5.7% of the human genome. Despite possessing constitutive heterochromatin features, these regions are transcribed to produce long noncoding RNAs with highly repetitive sequences that associate with specific sets of proteins to play various regulatory roles. In certain stress or pathological conditions, satellite RNAs are induced to assemble mesoscopic membraneless organelles. Specifically, under heat stress, nuclear stress bodies (nSBs) are scaffolded by HSat3 lncRNAs, which sequester hundreds of RNA‐binding proteins. Upon removal of the stressor, nSBs recruit additional regulatory proteins, including protein kinases and RNA methylases, which modify the previously sequestered nSB components. The sequential recruitment of substrates and enzymes enables nSBs to efficiently regulate the splicing of hundreds of pre‐mRNAs under limited temperature conditions. This review discusses the structural features and regulatory roles of satellite RNAs in intracellular architecture and gene regulation.

## Graphical abstract

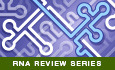

## Introduction

The human genome contains various types of repeat DNA regions. Among them, satellite DNA, estimated to constitute roughly 3% of the human genome, refers to long arrays of tandemly repeated sequences that are found in all centromeres and pericentromeric regions, and at specific locations in several chromosomes. The term “satellite DNA” originates from early biochemical experiments in the 1960s, in which genomic DNA was separated using cesium density gradient ultracentrifugation and an AT‐rich band was detected out of the major DNA band (Kit, 1961; Corneo *et al*, 1967). Restriction digestion methods also identified new classes of tandemly repeated sequences that could not be separated from the main genomic fraction on cesium density gradients (Manuelidis, 1976). In the human genome, satellite DNA is commonly AT rich, but the sequence and length of the repeat units vary among satellite DNA arrays, which can be classified into four main distinct classes. The largest class by total size is alpha satellite DNA (αSat), which encompasses the centromeres of all chromosomes (Manuelidis, 1976; Schueler *et al*, 2001). The large satellite arrays in the human genome are collectively termed classical human satellites, which comprise the three major classes of human satellites 1, 2, and 3 (HSat1–3). HSat1 can be further subdivided into two sequence families: a simple 42 bp tandem repeat (Prosser *et al*, 1986) and a 2.5 kbp repeat found predominantly on the Y chromosome (Frommer *et al*, 1984). Meanwhile, HSat2 and 3 are both derived predominantly from a tandem repeat of the pentamer “CATTC,” although HSat2 sequences appeared to be more divergent (Frommer *et al*, 1982; Prosser *et al*, 1986).

These satellite regions are usually embedded in highly condensed heterochromatin in interphase cells under normal physiological conditions. The role of αSat has been well characterized and has been shown to possess significant functions in the formation of the centromeric structure of chromosomes, which is required for kinetochore association during cell division (McKinley & Cheeseman, 2016; Altemose, 2022). The pericentromeric HSat DNAs are a platform for the formation of constitutive heterochromatin to organize inter‐chromosome interactions and/or locally alter gene expression, meiotic recombination, and nuclear architecture (Altemose, 2022).

Other than the roles of HSat regions in chromosomal maintenance and regulation, the HSat regions are transcribed under various stress conditions, in cancer cells, and in early embryonic and senescent cells to produce transcripts with repetitive RNA sequences (Jolly *et al*, 2004; Rizzi *et al*, 2004; Enukashvily *et al*, 2007; Valgardsdottir *et al*, 2008; Ting *et al*, 2011; Zhu *et al*, 2011, 2018; Bersani *et al*, 2015; Nogalski *et al*, 2019; Yandim & Karakulah, 2019; Nogalski & Shenk, 2020). Recent research has also shown that functional long noncoding RNAs (lncRNAs) are transcribed from the HSat regions. In particular, the lncRNAs transcribed from HSat2 and HSat3 play significant roles in the formation of phase‐separated, membraneless organelles (MLOs) by acting as structural scaffolds where they sequester multiple RNA‐binding proteins to regulate gene expression at transcriptional and post‐transcriptional levels under specific physiological conditions (Jolly *et al*, 2004; Rizzi *et al*, 2004; Hall *et al*, 2017; Ninomiya *et al*, 2020, 2021; Hirose *et al*, 2023). Based on the current knowledge of their roles, we propose naming transcripts encoded by HSat DNA regions “satellite RNAs” as a distinct subclass of lncRNAs and provide an account of the current understanding of the roles of satellite RNAs in gene regulation and MLO formation, together with their possible involvement in physiology and disease.

## Overview of human satellite DNAs in T2T genomic era

The human satellite DNAs, which were first characterized ~ 60 years ago, include αSat, HSat1, HSat2, and HSat3; they encompass the centromeric and pericentromeric chromosome regions with a size of several hundred megabases (Corneo *et al*, 1967; Prosser *et al*, 1986). However, owing to the highly repetitive nature of their sequences, most of these satellite regions have remained entirely missing from the human genome reference assembly in the 20 years since completion of the first assembly of genome sequencing data at the beginning of this century (Lander *et al*, 2001; Venter *et al*, 2001). Indeed, nearly all the centromeric and pericentromeric DNA sequences remain unassembled in the current GRCh38/hg38 reference sequence. This situation hindered research on satellite DNAs' functional and structural roles in the nucleus. The recent achievement of the first complete assembly of a human genome by the telomere‐to‐telomere (T2T) consortium opened a pathway toward understanding the roles of HSat1–3 (Altemose *et al*, 2022; Nurk *et al*, 2022). Here, we summarize the newly determined features of the HSat1–3 regions in T2T human genome data.

The T2T consortium reported a complete human genome assembly, including all autosomal αSat and HSat1–3 regions (Altemose *et al*, 2022; Nurk *et al*, 2022). To sequence the complete genome, the consortium chose to use PacBio HiFi and Oxford Nanopore ultralong read sequencing to assemble the uniformly homozygous CHM13hTERT cell line (Kronenberg *et al*, 2018; Nurk *et al*, 2020). This is a diploid cell line derived from a hydatidiform mole containing two copies of the paternal haplotype, making it useful for determining the sequence of highly repetitive genomic regions such as satellite DNA, whose sequences are often diverse due to mechanisms such as unequal crossover and gene conversion.

The complete genome sequence revealed that human centromeric and pericentromeric satellite DNAs represent 6.2% of the T2T‐CHM13v1.1 genome (~ 189.9 Mbp) (Altemose *et al*, 2022). All centromeric regions contain long tracts of tandemly repeated αSat arrays (total 85.2 Mbp, 2.8% of the genome). HSat1A constitutes a total of 13.4 Mbp (0.43% of the genome), mostly on chromosome 3 (chr3), chr4, and chr13, while HSat1B with 2.5 kbp repeats constitutes 14.2 Mbp on chrY and 1.2 Mbp on acrocentric chromosomes. HSat2 and HSat3 are 28.7 and 47.6 Mbp in total (0.94 and 1.56% of the genome), respectively, distributed on many chromosomes. Two additional large families, beta satellite (βSat) and gamma satellite (γSat), total 7.7 Mbp and 630 kbp, respectively, which are more GC rich than αSat and contain dense CpG methylation. The remainder are pericentromeric satellite DNAs (total 5.6 Mbp) and unresolved types of satellite DNA (total 1.2 Mbp; Altemose *et al*, 2022).

**Figure 1:**
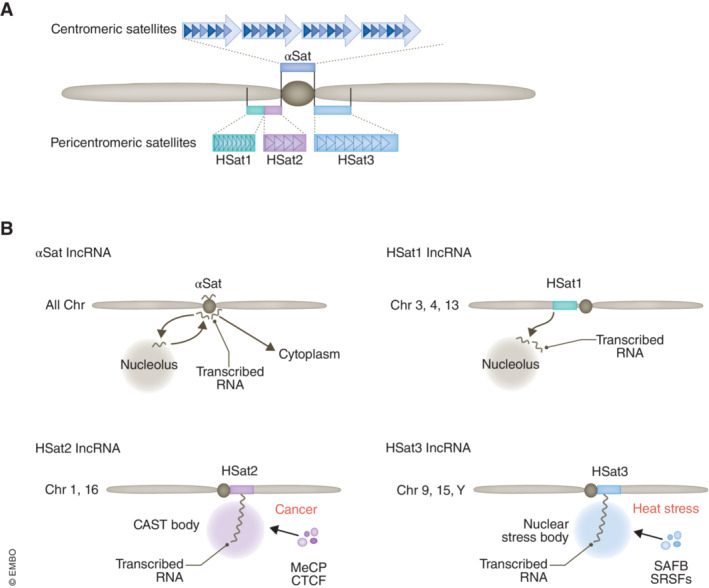
Summary of satellite DNAs and RNAs in human cells (A) Representative illustration of satellite DNAs in human chromosomes. Four types of satellite DNAs, namely αSat, HSat1, HSat2, and HSat3, are shown. Triangles represent repeat sequences comprising each satellite DNA. The αSat arrays are composed of HORs with different monomer repeat sequences repeated > 1,000 times. (B) Overview of satellite RNA functions. αSat RNAs are transcribed from centromeric αSat DNAs in all chromosomes. Intracellular localization of αSat lncRNAs is reported to be retained at the centromere, but another study reported the nucleolar localization of αSat lncRNAs, which relocate to the centromere at the beginning of mitosis. A further study also reported the centromeric localization of the minor population and the cytoplasmic localization of the major population. (B) HSat1A lncRNAs are thought to be mainly transcribed from the large HSat1A DNA and they are reported to colocalize with fibrillarin in the nucleolar periphery but not in the centromere. HSat2 lncRNAs are transcribed from HSat2 DNA, which includes large satellite regions in chr1 and chr16, particularly in cancer cells. HSat2 lncRNAs sequester proteins including MeCP and assemble MLOs termed CAST bodies. HSat3 lncRNAs are transcribed from HSat3 DNA, which includes a large satellite region in chr 9, under various stress conditions including heat stress. HSat3 lncRNAs sequester more than 100 proteins including SRSF1 and SAFB and assemble stress‐inducible MLOs termed nuclear stress bodies (nSBs).

Among these satellite DNAs, αSat expands to millions of bases composed of ~ 171 bp AT‐rich monomers. The largest αSat arrays are composed of different monomer subtypes called higher‐order repeats (HORs), which are large and highly homogeneous, and often contain thousands of nearly identical HOR units (Fig 1A). Kinetochore proteins only associate with a subset of these HOR units called active HORs. Pericentromeric HSat2 and HSat3 are derived from the (CATTC)n repeat sequence and constitute the largest contiguous arrays in the human genome (Fig 1A), including a 27.6 Mbp array on chr9, a 13.2 Mbp HSat2 array on chr1, a 12.7 Mbp HSat2 array on chr16, and a 7.5 Mbp HSat3 array on chr15. As for HSat1, a 5 Mbp array is found on chr13. The T2T‐CHM13 genome data constitute an enormous improvement over the hg38 reference assembly; the total HSat2 regions increase from 0.87 Mbp in hg38 to 28.7 Mbp in T2T‐CHM13, and the total HSat3 regions increase from 0.14 to 47.7 Mbp. Because chrY is not present in the CHM13 cell line, a T2T assembly of chrY from the HG002 cell line revealed that it includes 21.7 Mbp of HSat3 and 14.2 Mbp of HSat1B on chrY (preprint: Rhie *et al*, 2022).

## Chromatin features of satellite DNA regions

Satellite DNA regions form condensed heterochromatin with specifically modified histones and hypermethylated DNA that keep them transcriptionally silent. The structure of centromeric chromatin has a special feature associated with binding to a specific histone variant, which is essential for forming the functional centromeric structure. The centromeric αSat DNAs are significantly methylated and assemble specialized centromeric nucleosomes in which histone H3 is replaced by its variant, centromeric protein A (CENP‐A; Palmer *et al*, 1987). CENP‐A nucleosomes serve as the scaffold for the kinetochore that directly connects with spindle microtubules to mediate chromosome segregation (Westhorpe & Straight, 2014). A single human centromere contains only ~ 400 CENP‐A molecules (Bodor *et al*, 2014). CENP‐A nucleosomes are associated with CENP‐B, CENP‐C, and the CENP‐TWSX histone‐fold complex to form a centromere complex (Ando *et al*, 2002; Kato *et al*, 2013; Thakur & Henikoff, 2016).

Pericentromeric satellites such as HSat2 and HSat3 form condensed constitutive heterochromatin. The HSat heterochromatin loci are observed to be enriched in 5‐methylcytosine, which is implicated in condensation of pericentromeric chromosomes that prevents chromosomal segregation errors and is also related to aberrant DNA regulation in cell senescence (Swanson *et al*, 2013). The pericentric heterochromatin is characterized by the methylation of lysine 9 of histone H3 (H3K9me) and the trimethylation of lysine 20 of histone H4 (H4K20me3; Schotta *et al*, 2008). H3K9me3 on pericentromeric heterochromatin recruits the cognate reader protein heterochromatin protein 1 (HP1), which then recruits SUV4‐20H that in turn trimethylates H4K20 (Hahn *et al*, 2013). Despite the centromeric and pericentromeric satellites exhibiting typical heterochromatin features, they have been reported to be expressed under various stress conditions, developmental stages, and disease conditions such as in cancer cells. Human CENP‐B preferentially binds to unmethylated CENP‐B box DNA rather than the methylated form. However, CENP‐C, another component of the CENP‐A chromatin complex, is involved in repressing centromeric transcription (Gopalakrishnan *et al*, 2009). Human pericentromeric satellites are aberrantly expressed in various cancer cells, a result that coincides with the accumulation of methyl‐CpG‐binding protein 2 at pericentromeres (Hall *et al*, 2017).

## Satellite RNAs

Centromeric and pericentromeric satellite DNA repeats are actively transcribed during certain stages of the cell cycle, specifically transcribed upon certain stresses, and/or upregulated in disease‐related cells. Some of their transcripts are thought to be necessary for the formation and maintenance of heterochromatin and centromere function and some of the other conditionally expressed transcripts assemble MLOs by sequestering specific RNA‐binding proteins to regulate gene expression. This section particularly focuses on current knowledge of the features and functions of the RNA transcripts from human satellite DNAs including αSat, HSat1, HSat2, and HSat3 (Table 1).

**Table 1:** A summary of features and functions of satellite RNAs.

	αSat	HSat1	HSat2	HSat3
Chromosomes	All	HSat1A: 3, 4, 13, 14, 15, 21, 22	Major: 1, 2, 10, 16, Minor: Chr. 7, 15, 17, 22	Major: 9, 15, Y Minor: 1, 5, 10,13, 14, 17, 29, 21, 22
		HSat1B: Y		
Chromatin structure	Centrochromatin with CENPA nucleosomes	Constitutive heterochromatin	Constitutive heterochromatin	Constitutive heterochromatin
Sequence	171b monomer/higher‐order HOR arrays	HSat1A: 42 b monomer	23–26 b consensus tandem repeats	GGAAU repeats (with some variations)
		HSat1B: 2.5 kb		
Expression patterns	Detected in all cell cycle stages in various cell types	Vary between cancer cell lines and tissues	Induced by several stresses and in pathological conditions	Induced by various stresses, including thermal stress
RNA polymerase	RNA polymerase I, II, III	RNA polymerase II	RNA polymerase II	RNA polymerase II
Length	Various (1.3 kb specie was detected)	51–400 b	30–800 b	Various
Polyadenylation	Some of which are unlikely polyadenylated	Polyadenylated RNAs were detected	Likely non polyadenylated	Both nonpolyadenylated and polyadenylated RNAs were detected
Localization/MLO	Nucleolus, centromere, cytoplasm	Nucleolar periphery	CAST body	nSB
Binding proteins	INCENP, Aurora B, RBMX, DHX38	ND	MeCP2, CTCF, various DNA and RNA binding proteins	SAFBs, SRSFs, HNRNPs, m^6^A methylation complex
Molecular or physiological functions	Mitotic progression	ND	Cancer, aging	Regulation of transcription and splicing

## αSat lncRNA

αSat transcripts have been detected in multiple human cell types, although some inconsistent findings on their size, interactors, localization, and function have been reported (Wong *et al*, 2007; Chan *et al*, 2012; Ideue *et al*, 2014; Quenet & Dalal, 2014; McNulty *et al*, 2017). RNA polymerase II (RNAPII) is implicated in the transcription of human αSat DNA, which is based on observations of the centromeric localization of RNAPII and the effect of RNAP inhibitors on transcript accumulation. RNAPI and RNAPIII are also reported to influence the expression and localization of αSat transcripts (Wong *et al*, 2007; Klein & O'Neill, 2018). The slow turnover of repetitive RNAs has been documented and suggests that aSat RNAs are innately stable (Hall *et al*, 2014; McNulty *et al*, 2017). It has been suggested that at least some αSat RNAs lack poly‐A tails (Ideue *et al*, 2014). It remains to be elucidated whether the stability of αSat RNAs is conferred by the presence of a poly‐A tail or another protective mechanism. Post‐transcriptional processing of α‐Sat transcripts such as capping and splicing has yet to be investigated.

Centromeres are known to cluster around nucleoli in mammalian cells, but the significance of the nucleolus–centromere interaction remains poorly understood. αSat RNAs were reported to initially localize in the nucleolus and relocalize to the centromere at the onset of mitosis (Wong *et al*, 2007). Quantitative analysis of αSat RNAs by single‐molecule fluorescent *in situ* hybridization (smFISH) revealed that the levels of αSat RNA‐smFISH foci vary across cell lines and over the course of the cell cycle. Notably, the proportion of nucleolar‐localized centromeres inversely correlates with αSat RNA levels across cell lines and the αSat RNA levels increase substantially when the nucleolus is disrupted, suggesting that the centromere–nucleolus interactions suppress αSat expression (Bury *et al*, 2020). Monitoring of the nucleolus–centromere interactions revealed dynamic changes in these interactions during cell differentiation and in cancer cells, which correlated with changes in nucleolar characteristics (Rodrigues *et al*, 2023). This raises the intriguing possibility that the dynamic changes in the nucleolus–centromere interactions are regulated by the conditional synthesis and accumulation of αSat RNAs in various cellular contexts. Meanwhile, αSat has also been reported to localize to centromeres in both interphase and metaphase (Ideue *et al*, 2014; Quenet & Dalal, 2014; McNulty *et al*, 2017), where it colocalizes with centromere proteins. smFISH also showed that the majority of αSat RNAs do not remain associated with centromeres (Bury *et al*, 2020): In interphase cells, αSat RNAs were localized in the nucleus but only ~ 10% of smFISH foci overlapped with centromeres. In addition, in mitotic cells, smFISH foci did not associate with chromatin, and αSat RNAs appeared broadly distributed within the cytoplasm and disappeared as cells passed through cytokinesis (Fig 1B).

αSat transcripts have been shown to form complexes with centromere proteins CENP‐A and CENP‐C, as well as CENP‐B, which also binds αSat DNA (Quenet & Dalal, 2014; McNulty *et al*, 2017). αSat RNAs of 1.3 kb in size physically interact with the soluble pre‐assembly HJURP/CENP‐A complex prior to association with centromeric chromatin (Quenet & Dalal, 2014). The αSat RNAs are transcribed by RNAPII at late mitosis into early G1 concurrent with the timing of new CENP‐A assembly. Inhibition of RNAPII transcription abrogates the recruitment of CENP‐A and its chaperone HJURP to native human centromeres. Knockdown of αSat RNA using an shRNA led to mitotic defects and reduced CENP‐A loading, implying an essential role for αSat RNAs in centromere function (Quenet & Dalal, 2014).

Single‐stranded αSat transcript is also suggested to bind SUV39H1 histone lysine methyltransferase involved in K9 trimethylation in heterochromatin (Johnson *et al*, 2017). αSat RNAs may recruit SUV39H to the pericentromeric region to form constitutive heterochromatin in human cells because mutant SUV39H lacking nucleic acid binding ability prevented its association with heterochromatin (Johnson *et al*, 2017). αSat RNA might recruit HP1 as a reader of K9 trimethylation in human cells because HP1 requires RNA to localize to pericentromeric chromatin in mouse cells (Maison *et al*, 2011). These findings are consistent with a previous report pointing to RNA component(s) being involved in pericentric heterochromatin maintenance, which was based on the observation that RNase treatment resulted in structural alteration of the pericentromeric heterochromatin (Maison *et al*, 2002).

## HSat1 lncRNA

There had long been a lack of studies on the transcripts from HSat1 regions, but recently Lopes *et al* (2023) extensively characterized the expression and intracellular localization of transcripts from HSat1A regions, which are located mostly on chr3, chr4, and chr13. HSat1 expression levels vary markedly in various cancer cell lines and human tissues. The copy numbers of HSat1 DNA monomers in the genome are also highly variable, but little correlation between genomic copy numbers and RNA expression levels has been detected. Physical mapping of HSat1 lncRNA in the human chromosomes by FISH revealed clear FISH signals in all acrocentric chromosomes and chromosomes 1 and 3 (Lopes *et al*, 2023). RNA‐FISH showed that HSat1A transcripts exhibit cluster‐like organization in the nucleus, albeit with distinct signal patterns in different cell lines. HSat1A RNA‐FISH coupled with immunofluorescence (IF) of the nucleolar protein fibrillarin showed that HSat1A transcripts are spotted contiguously to the nucleolar periphery (Fig 1B). DNA and RNA signals of HSat1A have been shown to be differently arranged in DNA/RNA FISH experiments, even though putative nascent RNA signals colocalize with HSat1A DNA (Lopes *et al*, 2023). Oligo‐dT primed 3′ RACE (rapid amplification of cDNA ends) with a forward PCR primer targeting the HSat1A sequence suggested that HSat1A transcripts are polyadenylated and thus likely transcribed by RNAPII. Meanwhile, ~ 70% of these HSat1A transcripts were distributed across a size range of 51 to ~ 400 b, with peaks corresponding to multiples of the 42 b monomer size, which are likely produced by alternative polyadenylation (Lopes *et al*, 2023).

## HSat2 lncRNAs

HSat2 is a primate‐specific satellite repeat located at pericentromeric regions of subsets of chromosomes. The largest arrays reside within the pericentromeres of human chromosomes 1 and 16, while small blocks of HSat2 sequences are found in pericentromeres of human chromosomes 2, 5, 7, 10, 13–15, 17, 21, 22, and Y (Richard *et al*, 2008; Hall *et al*, 2017). In human chromosome 1q12, HSat2 is colocalized with the HSat3 region (Porokhovnik *et al*, 2021). HSat2 DNA consists of divergent variants of 23–26 bp consensus tandem repeats (Bersani *et al*, 2015). While transcription of HSat2 is normally silenced, it is highly induced in various types of cancers, facioscapulohumeral muscular dystrophy (FSHD), immunodeficiency centromeric heterochromatin, facial anomalies immunodeficiency syndrome, and by herpesvirus infection, senescence, and DNA damage response (Hassan *et al*, 2001; Ting *et al*, 2011; Cruickshanks *et al*, 2013; Nogalski *et al*, 2019; Shadle *et al*, 2019; Nogalski & Shenk, 2020; Miyata *et al*, 2021). HSat2 RNA is highly expressed in various cancers, including pancreatic cancer, and is also expressed in preneoplastic pancreatic lesions, suggesting that it could serve as a biomarker for pancreatic cancer (Ting *et al*, 2011). In addition, HSat2 copy number gain is commonly observed in human colon tumors (Bersani *et al*, 2015). HSat2 RNAs are exported to the extracellular space via extracellular vesicles (Miyata *et al*, 2021). HSat2 RNA is also found in the circulating blood of cancer patients; accordingly, a sensitive detection system has been developed to identify this biomarker (Kishikawa *et al*, 2016). Furthermore, HSat2 RNA is induced in the genetic disorder FSHD due to the abnormal expression in muscles of the transcription factor DUX4, the binding sequence of which closely matches the consensus sequences of HSat2 (Shadle *et al*, 2019). These reports suggest that HSat2 RNAs are associated with various pathological conditions. However, the basic features of HSat2 RNAs remain largely uncharacterized. Regarding the size of these transcripts, northern blotting generates a pattern of bands ranging from 30 to > 800 b in size (Bersani *et al*, 2015). Although direct evidence is lacking, RNAPII likely generates HSat2 RNAs, at least in the case of DUX4‐induced conditions (Shadle *et al*, 2019). Analyses using poly(A)‐selected and total RNA‐seq have suggested that HSat2 RNAs lack poly(A) tails (Solovyov *et al*, 2018); however, to the best of our knowledge, no direct evidence of HSat2 RNAs possessing a 5′‐cap, introns, and RNA modifications has been presented.

HSat2 lncRNAs form nuclear MLOs, called CAST (cancer‐associated satellite transcript) bodies, around their transcription sites (Fig 1B; Hall *et al*, 2017; Smith *et al*, 2020). Exogenous HSat2 expression in cells not expressing HSat2 leads to the formation of CAST bodies, suggesting an architectural role of HSat2 in the formation of these nuclear bodies (Landers *et al*, 2021). HSat2 lncRNAs interact with many nuclear proteins including MeCP2 (Methyl CpG‐binding protein 2) and CTCF (Hall *et al*, 2017; Miyata *et al*, 2021). These CAST bodies act as molecular sponges to sequester master epigenetic regulatory proteins, including MeCP2, and are thought to influence the epigenome in cancer cells. Through the HSat2–CTCF interaction, the CAST body sequesters CTCF and regulates chromatin (Miyata *et al*, 2021). In addition to HSat2 RNAs, unmethylated HSat2 DNA, forms nuclear foci called cancer‐associated polycomb (CAP) bodies from the 1q12 mega‐satellite, which also act as molecular sponges to sequester the polycomb group complex‐component PRC1 (Hall *et al*, 2017). These sequestrations cause epigenetic instability, which is recognized as a hallmark of cancer (Hall *et al*, 2017). In addition, cells stably overexpressing HSat2 exhibit defects in cell division, which is another hallmark of cancers (Landers *et al*, 2021). In FSHD, CAST bodies sequester double‐stranded RNAs, and EIF4A3 and ADAR1 proteins, which may underlie the pathogenic mechanism (Shadle *et al*, 2019). Although many reports have been published on the roles of HSat2 RNAs in pathological conditions, the specific physiological function of CAST bodies remains unknown.

## HSat3 lncRNAs

**Figure 2:**
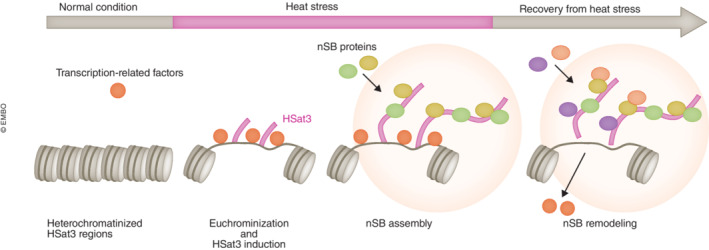
Schematic presentation of HSat3 induction and nSB assembly HSat3 regions are in the form of heterochromatin under normal conditions and euchromatin upon heat stress. Transcription factors accumulate at HSat3 regions, and HSat3 lncRNAs are thereby transcribed and recruit specific RBPs. After stress removal, remodeling of nSB components occurs (see also Table 2). The green and yellow circles indicate constitutive nSB proteins and the purple and beige circles indicate recovery phase‐specific nSB proteins.

HSat3 is a primate‐specific satellite repeat located at pericentromeric regions in subsets of chromosomes. The large arrays (3.5–27.6 Mbp) reside within the pericentromeres of human chr9, chr15, and chrY. HSat3 regions with (CATTC)n repeat sequences form transcriptionally silent heterochromatin under normal conditions; however, they are converted into transcriptionally active euchromatin rich in acetylated histones upon exposure to heat stress (Fig 2; Jolly *et al*, 2004; Rizzi *et al*, 2004). In this situation, heat shock transcription factor 1 (HSF1), which is activated upon heat shock, promotes HSat3 transcription by RNAPII, probably by recognizing heat shock responsive element (HSE)‐like sequences scattered in the HSat3 genomic sequence (Jolly *et al*, 2002). HSat3 transcription occurs unidirectionally from the G‐rich strand to produce HSat3 lncRNA with a GGAAU repeat sequence (Jolly *et al*, 2004) (Valgardsdottir *et al*, 2005). HSat3 transcription has also been reported to be induced upon other types of stresses such as osmotic stress and UV irradiation, and by treatment with chemical compounds such as etoposide (Valgardsdottir *et al*, 2008). Transcribed HSat3 lncRNAs accumulate at transcription sites on the HSat3 chromosome regions where they dedicate themselves to assembling MLOs called nuclear stress bodies (nSBs; Fig 1B). HSat3 lncRNAs mainly comprise the GGAAU repeat sequence; however, they are heterogeneous transcripts that differ in length and sequence. Because of their repetitive nature, HSat3 lncRNA sequences have only partially been determined. Even the average length of HSat3 lncRNAs is still controversial; some reports showed that the length of HSat3 lncRNAs is in the range 2–5 kb (Rizzi *et al*, 2004; Valgardsdottir *et al*, 2005), while another report showed that HSat3 RNAs are too long to migrate from the top of the gel in agarose gel electrophoresis (Jolly *et al*, 2004). One of the reasons for the limited knowledge about the precise sequences is that HSat3 lncRNAs are transcribed from multiple sites of the pericentromeric regions of various chromosomes. In addition, HSat3 transcription is believed to start from multiple HRE sites scattered in each HSat3 region. Some reports published in the 2000s suggested that HSat3 lncRNAs are polyadenylated at the 3′ end because they were detected in RNA fractions purified using an oligo‐dT column (Rizzi *et al*, 2004). In contrast, we confirmed that the majority of HSat3 lncRNAs remained in the oligo‐dT unbound (poly(A)‐RNA) fraction (our unpublished data). Therefore, the typical 3′ end structure of HSat3 lncRNAs is still obscure. Various splicing factors and small nuclear ribonucleoproteins (snRNPs) are associated with HSat3 lncRNAs, as described later (Metz *et al*, 2004), implying the possibility that Hsat3 lncRNAs undergo splicing within nSBs. However, whether Hsat3 transcripts contain introns remains unclear.

Hsat3 lncRNAs associate with specific sets of RNA‐binding proteins (RBPs) including scaffold attachment factor B (SAFB), SR splicing factors (SRSFs), and heterogeneous nuclear ribonucleoproteins (HNRNPs), which results in the formation of massive nSBs (Denegri *et al*, 2001; Biamonti & Caceres, 2009). nSBs remain stable in the nucleus for several hours even after the stress has been removed and the HSat3 transcription is attenuated. Heat shock‐exposed cells exhibit multiple nSB foci of various sizes. The size of each nSB focus is generally considered to depend on the size of the HSat3 region of each pericentromeric region. Given that nSBs disappear upon HSat3 knockdown (KD) and ectopic transformation of human chromosome 9, 12, or 15 in nonprimate cells recapitulates nSB formation (Denegri *et al*, 2002), HSat3 lncRNAs are clearly essential and sufficient for nSB assembly. Thus, HSat3 lncRNAs can be considered structural scaffolds of nSBs. A number of lncRNAs have been reported to play similar roles as structural scaffolds of specific MLOs, therefore “architectural RNA” (arcRNA) has been proposed as a functional lncRNA category (Chujo & Hirose, 2017; Yamazaki *et al*, 2019). More than 10 arcRNAs have been reported in various eukaryotic species. For example, NEAT1_2, HSRω, and Mei arcRNAs act as structural scaffolds of nuclear paraspeckles in mammals, ω‐speckles in fly, and Mei2 dots in fission yeast, respectively (Watanabe & Yamamoto, 1994; Prasanth *et al*, 2000; Sasaki *et al*, 2009; Chujo & Hirose, 2017; Yamazaki *et al*, 2019). HSat2 lncRNA would be categorized as an arcRNA for the assembly of CAST bodies (Hall *et al*, 2017), although further characterization is required to include it on the list of arcRNAs. HSat3 is a model arcRNA for investigating the molecular mechanism of the arcRNA‐dependent assembly of MLOs. nSBs have been studied for their components and mode of action to regulate heat stress‐responsive gene expression. The following section particularly focuses on recent insights into the structure and function of nSBs.

## Basic features and components of nSBs

nSBs were first described as perichromatin granules (PGs) acting as nuclear sites for the accumulation of HNRNPs (Mahl *et al*, 1989). Later, activated HSF1 was observed to localize in a few nuclear foci with a maximum diameter of 2–2.5 μm under heat stress (Sarge *et al*, 1993). nSBs are detected in human and other primate cells, but not in mouse cells. The number of nSBs varies among cell lines in a manner that correlates with cell ploidy. The importance of RNA for nSB structural integrity was shown by the observation that nSBs are sensitive to RNase treatment (Chiodi *et al*, 2000). In 2002, two groups reported that nSBs are formed on the HSat3 chromosomal region and HSat3 transcripts are structural scaffolds of nSBs (Denegri *et al*, 2002; Jolly *et al*, 2002). Consistent with this, nSBs formed on RNA scaffolds have been shown to contain multiple RNA‐binding proteins such as SAFB and some SRSFs (Jolly & Morimoto, 1999; Denegri *et al*, 2001) (Biamonti & Vourc'h, 2010). A recent comprehensive analysis by HSat3‐chromatin isolation by RNA purification‐mass spectrometry (ChIRP‐MS) revealed 141 HSat3 RBPs as candidates for nSB components. Most of these are RBPs and their modifiers associated with RNA processing, such as pre‐mRNA splicing, m^6^A RNA modification, and RNA nuclear export (Ninomiya *et al*, 2020). nSBs also contain transcription‐related factors and chromatin remodeling factors, such as HSF1, CREB‐binding protein (CBP), BRG1, and bromodomain protein 4 (BRD4) (Kawaguchi *et al*, 2015; Goenka *et al*, 2016; Hussong *et al*, 2017). Because these factors were not detectable by HSat3‐RNA ChIRP, they most likely interact with HSat3 DNAs, not directly with HSat3 RNAs. nSBs have also been suggested to contain other RNAs such as U1 snRNA and initiator tRNA (Metz *et al*, 2004; Miyagawa *et al*, 2012; Ninomiya *et al*, 2021). Notably, nSBs exhibit dynamicity by changing their protein components in a temperature‐ and/or time course‐dependent manner (Fig 2 and Table 2; Jolly *et al*, 2004;Ninomiya *et al*, 2020, 2021). Transcription‐related factors such as HSF1, CBP, and RNAPII are detectable within nSBs during heat shock but removed from nSBs after the removal of this stress (Jolly *et al*, 2004), presumably reflecting the attenuation of HSat3 transcription after stress removal. In contrast, RNA/RBP‐modifying enzymes such as CLKs, a family of nuclear SR protein kinase and RNA m^6^A modification factors, are recruited into nSBs mainly after stress removal (Ninomiya *et al*, 2020, 2021). Such dynamics of the nSB components are inseparable from the molecular functions of nSBs described later. Another significant feature of nSB components is that nSBs are classified into at least two subclasses by the distinct sets of colocalizing RBPs: a major subclass including SAFB (named nSB‐S) and a minor population of nSBs including HNRNPM (named nSB‐M) (Aly *et al*, 2019). Under electron microscopy, SAFB localizes at a highly dense cluster of multiple PGs, whereas HNRNPM localizes adjacent to SAFB in the core of the PGs (Chiodi *et al*, 2000).

**Table 2:** Major protein components of nSBs.

	nSB components	Putative roles in nSBs
Constitutive	Acetylated histone H4	Eu chromatinization of HSat3 region
	SRSFs	Splicing regulation
	SR‐related factors	Splicing regulation
	HNRNPs	Unknown
	RNA export factors	Unknown
	SAFB1/2 and SLTM	Unknown
Heat shock phase	HSF1	Promotion of HSat3 transcription
	RNAPII	Transcription of HSat3 lncRNAs
	CBP (CREBBP)	Sequestrated within nSBs to repress transcription of target genes
Recovery phase	CLK1 SR kinase	Rephosphorylation of SRSFs
	m^6^A writer complex	m^6^A modification of HSat3 lncRNAs
	YTHDC1 m^6^A reader	Sequestrated within nSBs resulting in repression of nucleoplasmic m^6^A‐dependent pre‐mRNA splicing

## Mode of nSB actions to regulate temperature‐dependent pre‐mRNA splicing

**Figure 3:**
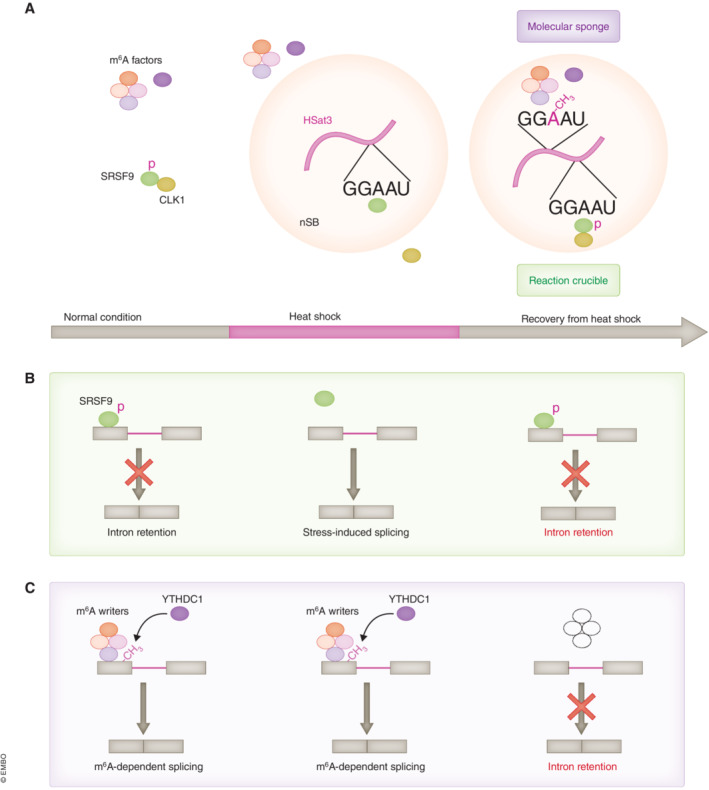
Two modes of nSB action for regulating splicing (A) Overview of biochemical modifications within nSBs. HSat3 lncRNAs concentrate dephosphorylated SRSFs within nSBs during heat stress and recruit CLK1 kinase into nSBs after stress removal to rapidly rephosphorylate SRSFs (reaction crucible). HSat3 lncRNAs also recruit m^6^A writer complex after stress removal to undergo RNA m^6^A modification, thereby sequestering an m^6^A reader protein, YTHDC1 (molecular sponge). (B) Through the action as the reaction crucible, nSBs rapidly normalize the heat stress‐induced dephosphorylation of SRSFs and splicing alterations after stress removal. (C) As molecular sponges, nSBs sequester m^6^A factors, resulting in inhibition of the m^6^A modification of other pre‐mRNAs and their m^6^A‐dependent splicing.

Under heat stress, HSat3 lncRNAs are reported to modulate the transcription of specific genes and also control the splicing of hundreds of pre‐mRNAs by promoting nuclear intron retention, mainly during the recovery from the stress (Goenka *et al*, 2016; Ninomiya *et al*, 2020). Nuclear intron retention (also referred to as “intron detention” to avoid confusion with classical intron retention) is a recently defined type of splicing regulation, in which partially unspliced pre‐mRNAs retaining specific intron(s) are pooled in the nucleus and undergo post‐transcriptional splicing of the retained intron(s) to produce mature mRNAs, in some cases upon certain stimuli (Ninomiya *et al*, 2011; Boutz *et al*, 2015; Mauger *et al*, 2016). Recent reports showed that HSat3 lncRNAs control these splicing events through two modes of action via biochemical modifications of RBPs and RNA that occur within nSBs at specific times during the recovery from stress (Fig 3A; Ninomiya *et al*, 2020, 2021).

The first mode of nSB action is the “reaction crucible mechanism.” In this mechanism, nSBs concentrate specific sets of SRSFs and SR‐related proteins, most of which are likely dephosphorylated, during heat stress exposure, and then nSBs recruit CLK1, a nuclear protein kinase that phosphorylates SRSFs and SR‐related proteins, immediately after stress removal. The sequential recruitment of dephosphorylated SRSFs as substrates and CLK1 as an enzyme enables nSBs to serve as a platform to rapidly rephosphorylate SRSFs, especially SRSF1 and SRSF9 (Ninomiya *et al*, 2020). In turn, the rephosphorylated SRSFs regulate splicing of more than 100 pre‐mRNAs. In this context, nSBs act as reaction crucibles for phosphorylation reactions of specific confined substrates under specific temperature conditions. CLK1 is recruited to nuclear speckles (NSs), other MLOs containing splicing‐related factors that partially overlap with those of nSBs, both during and after heat shock. The temperature‐dependent regulation of CLK1 recruitment is only observed in nSBs. Heat shock‐induced dephosphorylation of SRSFs triggers global splicing arrest and alternative splicing (Shin *et al*, 2004; Biamonti & Caceres, 2009; Ninomiya *et al*, 2011). In this context, HSat3 lncRNAs may contribute to rapid normalization of heat shock‐induced splicing alterations after removal of the stress (Fig 3B).

The second mode of action is the “molecular sponge mechanism.” In this mechanism, HSat3 lncRNAs recruit m^6^A writer complex, including METTL3 and WTAP, mainly during recovery from the stress (Fig 3A). The adenosines in the GGAAU repeat of HSat3 lncRNAs are partially m^6^A modified (~ 10% of total adenosines in GGAAU repeat of Hsat3), resulting in marked enrichment of m^6^A‐modified RNAs in nSBs. This enables nSBs to sequester nuclear m^6^A reader protein YTHDC1 from the surrounding nucleoplasm. Consequently, m^6^A modification levels of nucleoplasmic pre‐mRNAs are diminished and the YTHDC1‐dependent splicing of these pre‐mRNAs is suppressed to accumulate intron‐retained pre‐mRNAs in the nucleus (Fig 3C; Ninomiya *et al*, 2021). These findings delineate the distinct function of nSBs as molecular sponges of m^6^A factors through m^6^A modification of the scaffolded HSat3 lncRNAs. Thus, two distinct mechanisms regulate the splicing of distinct but not mutually exclusive sets of nSB target introns. Interestingly, some of the HSat3 target introns are intensively controlled by both mechanisms in nSBs. nSBs also concentrate HNRNPs, which are generally thought to antagonize SRSFs, suggesting that HSat3 lncRNAs may regulate splicing through an additional mechanism mediated by the HNRNPs. The regulatory functions of nSBs as reaction crucibles and molecular sponges to control RNA processing raise the intriguing possibility that HSat3 DNA and/or RNA also regulate transcription by concentrating or sequestering chromatin remodeling factors, transcription factors, and RNAPII.

Most canonical RBPs recognize 5–6‐nucleotide (nt) motifs for specific RNA binding (Ray *et al*, 2013; Dominguez *et al*, 2018). Therefore, arcRNAs possessing short tandem repeats of 5–6 nt are generally thought to be optimal for highly concentrating specific RBPs (Yap *et al*, 2018; Ninomiya & Hirose, 2020). Among such short‐tandem repeat RNAs, HSat3 lncRNAs are unique in having GGAAU repeats that are compatible with binding to both SRSF9 and m^6^A factors. The GGAAU repeat does not perfectly match the m^6^A consensus GGACU motif (the C is essential), and the binding efficiency of m^6^A writer proteins with GGAAU repeat is weaker than that with GGACU repeat. However, a notably high number of GGAAU repeats in HSat3 likely enables the sequestration of m^6^A factors at a level sufficient to competitively repress m^6^A modification of other nucleoplasmic pre‐mRNAs. Meanwhile, SRSF9 preferentially binds GGAAU repeats, but cannot bind m^6^A‐modified GGAAU repeats *in vitro* (Ninomiya *et al*, 2021). Therefore, the low efficiency of m^6^A modification allows HSat3 lncRNAs to properly recruit SRSF9 and m^6^A reader proteins via the unmethylated and methylated regions, respectively.

## Possible roles of nSBs in physiological events and diseases

HSat3 lncRNAs are expressed to form nSBs under various physiological conditions including stress, disease, and senescence, suggesting their regulatory roles in each of these conditions. They are also suggested to protect cells from heat shock‐induced apoptosis (Goenka *et al*, 2016; Watanabe & Ohtsuki, 2021). Meanwhile, HSat3 lncRNAs have also been suggested to behave as toxic RNAs by causing irregular mitotic segregation of chromosomes a few days after the removal of stress (Giordano *et al*, 2020). In this case, HSat3 lncRNAs are suggested to be fragmented into 25–75 b small RNAs, which are associated with anomalously segregated chromatin. Considering that the cell cycle is arrested during heat shock exposure and for several hours after stress removal, HSat3 lncRNAs somehow exhibit protective effects against heat shock‐induced apoptosis during heat shock and during the early phase of recovery, but residual HSat3 lncRNAs and/or their fragmented small RNAs may be toxic to the cells after the cell cycle resumes. In either case, the molecular basis of the cellular roles of HSat3 lncRNAs remains largely unknown.

Spinocerebellar ataxia type 31 (SCA31) is a neurological disease caused by HSat3‐like GGAAU repeat RNA. Intron 6 of brain expressed associated with NEDD4‐1 (*BEAN1*) gene contains around 20 repeats of TGGAA, which expands to > 200 repeats in SCA31 patients (Sato *et al*, 2009; Niimi *et al*, 2013; Ishiguro *et al*, 2017).

**Figure 4:**
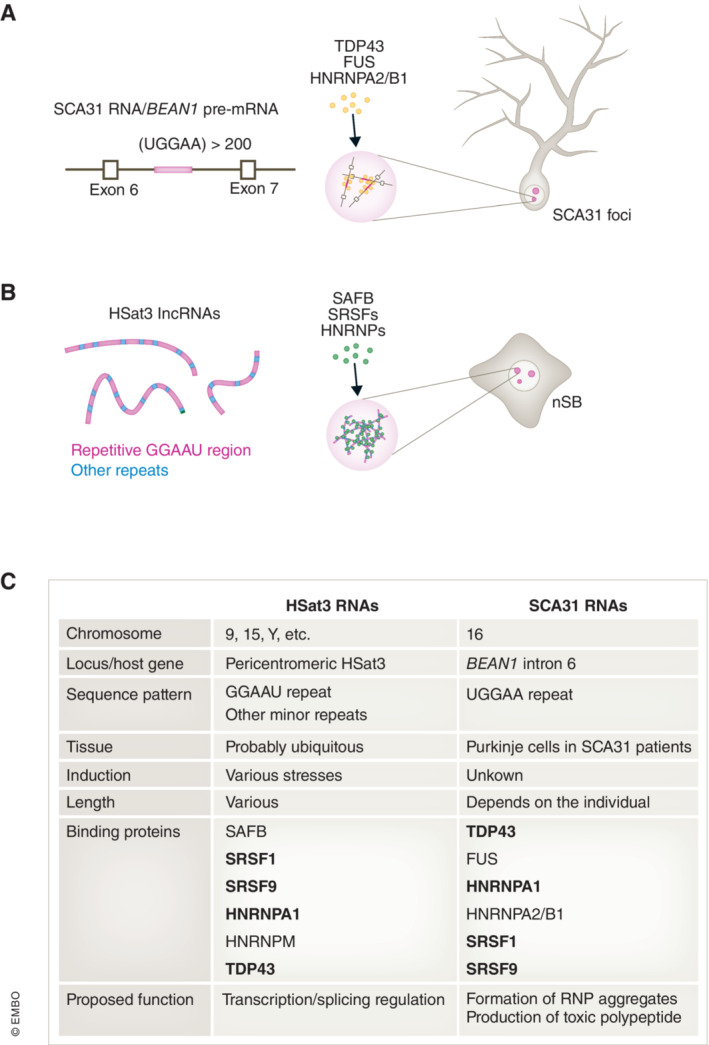
Comparison of SAC31 foci and nSBs (A, B) Schematic illustration of SCA31 foci (A) and nSB (B). (C) List of features of HSat3 and SCA31 RNA. The common binding proteins are shown in bold.

The expanded repeat form of BEAN1 was named SCA31 RNA because it is the pathogenic RNA for SCA31. SCA31 RNA accumulates in the nucleus to form RNA foci in Purkinje cells of the cerebellum of SCA31 patients (Fig 4A and C). The UGGAA tandem repeat in SCA31 RNA is equivalent to the major GGAAU repeat in HSat3 lncRNAs, although HSat3 RNAs also contain other repeat sequences at low frequency. Therefore, nSBs and SCA31 foci likely share some structural and functional features (Fig 4B and C). For example, interactome analyses of HSat3 lncRNA and SCA31 RNA revealed that these RNAs interact with common RBPs such as SRSF9 and TDP43 (Fig 4C; Sato *et al*, 2009; Niimi *et al*, 2013; Ishiguro *et al*, 2017). Naphthyridine carbamate dimer (NCD) is a synthetic small compound that preferentially binds UGGAA/GGAAU repeat and prevents interactions with several UGGAA‐binding RBPs *in vitro* (Shibata *et al*, 2021). NCD also prevents the assembly of SCA31 foci in cells and thereby alleviates SCA31 symptoms in the disease model fly (Shibata *et al*, 2021). NCD also prevents nSB assembly and nSB function in splicing regulation (Shibata *et al*, 2021). Considering the similar features of nSBs with disease‐relevant SCA31 foci and the advantage that nSBs are easily and rapidly inducible upon heat stress, nSBs can be a useful evaluation model for screening chemicals with therapeutic potential for SCA31 disease. In addition to SCA31, repeat sequence RNAs sequester specific RBPs in a manner dependent on the repeat sequence and cause various neurological diseases (Malik *et al*, 2021). Basic research on the mode of action of nSBs would provide valuable mechanistic insights into the onset of these diseases and possible therapeutic targets involved in their mechanisms.

## Concluding remarks

Satellite DNAs are considered a major source of heterochromatin in the nucleus, but it has been revealed that each class of satellite DNA is transcribed to produce satellite RNA under specific physiological and/or pathological conditions. This is in turn suggested to play various regulatory roles in gene expression. Each class of satellite RNAs is a mixture of heterogeneous RNAs with a common major repeat sequence. The difficulty in obtaining precise nucleotide sequences of satellite RNAs using a canonical short‐read sequencer owing to their repetitiveness has hindered research on the role of specific satellite RNAs. T2T genome sequencing would facilitate precise mapping of the transcription start and termination sites of each satellite RNA using a long‐read sequencer, which would enable the roles of each satellite RNA molecule to be distinguished. To understand how low‐complexity repeat sequences work, a synthetic biology approach using artificially designed RNA sequences can be effective. For example, the artificial expression of specific disease‐related repeat sequence RNAs can induce the formation of MLOs by sequestering specific RBPs (Ishiguro *et al*, 2017), suggesting that this kind of approach would be applicable to investigating the mode of action of specific satellite RNAs. Antisense oligonucleotide (ASO)‐mediated precipitation of satellite RNA–protein complexes such as by the ChIRP method has been shown to be a powerful tool for extensively identifying the associating proteins since a single ASO highly efficiently captures RNP complexes. Identification of the components of satellite RNPs also deepens our understanding of the functions of satellite RNAs. In addition to those in humans, satellite RNAs have been reported in various eukaryotic species such as mouse, fly, and nematode (Probst *et al*, 2010; Burton & Torres‐Padilla, 2014; Rosic *et al*, 2014; Mills *et al*, 2019; Subirana & Messeguer, 2023). In early mouse development, major satellite repeat (MSR) transcription normally occurs, but its failure results in defective heterochromatin establishment and abrogation of embryo development (Probst *et al*, 2010; Burton & Torres‐Padilla, 2014). MSRs are highly transcribed in mouse embryonic stem cells (ESCs). The MSR RNAs can drive the formation of HP1 droplets *in vitro* and also modulate the biophysical properties of heterochromatin, allowing dynamic condensates to form in ESCs. Depletion of MSR RNAs causes heterochromatin to transition into a more compact and static state, which has severe consequences for ESCs, including chromosome instability and mitotic defects (Novo *et al*, 2022). This highlighted the essential role of satellite RNAs in modulating the organization and properties of heterochromatin in addition to the formation of nuclear MLOs. Considering that unique satellite sequences have been acquired in a clade‐specific manner, satellite RNAs may have played an important role in biological evolution, including speciation (Thakur *et al*, 2021). Additionally, given that satellite sequence length has been reported to differ among individuals, the differing functions of satellite RNAs expressed from these sequences may contribute to individual physiological differences (Liehr, 2021). Thus, by advancing our understanding of the function of satellite RNAs, we can expect to gain insights that would resolve biological problems from a new perspective.

## Author contributions

**Kensuke Ninomiya:** Conceptualization; funding acquisition; writing – original draft; writing – review and editing. **Tomohiro Yamazaki:** Conceptualization; funding acquisition; writing – original draft; writing – review and editing. **Tetsuro Hirose:** Conceptualization; supervision; funding acquisition; writing – original draft; project administration; writing – review and editing.

## Disclosure and competing interests statement

The authors declare that they have no conflict of interest.
